# *N*′-(3-Methyl­quinoxalin-2-yl)-*N*′-phenyl­benzohydrazide

**DOI:** 10.1107/S160053681101703X

**Published:** 2011-05-11

**Authors:** Youssef Ramli, Ahmed Moussaif, Hafid Zouihri, Houda Bourichi, El Mokhtar Essassi

**Affiliations:** a Laboratoire Nationale de Controle des Médicaments, Direction du Médicament et de la Pharmacie, BP 6206, 10000 Rabat, Morocco; b Laboratoire de Chimie Hétérocyclique, Pole de Compétence PHARCHIM, Université Mohammed V-Agdal, BP 1014, Rabat, Morocco; c Unité de la Radioimmunoanalyse, Centre National d’Etudes Scientifiques et Techniques d’Energie Nucléaire, Maamoura, Morocco; d Laboratoire de Diffraction des Rayons X, Division UATRS, Centre National pour la Recherche Scientifique et Technique, Rabat, Morocco

## Abstract

In the crystal structure of the title compound, C_22_H_18_N_4_O, the quinoxaline system makes dihedral angles of 86.59 (7) and 63.37 (9)° with the benzohydrazide and phenyl rings, respectively. The benzohydrazide ring makes a dihedral angle of 72.46 (10)° with the phenyl ring. The crystal structure is stabilized by inter­molecular N—H⋯O hydrogen bonds, C—H⋯O contacts and C—H⋯π inter­actions.

## Related literature

For the biological activity of quinoxaline derivatives, see: Kleim *et al.* (1995 ▶). For the anti­tumour and anti­tuberculous properties of quinoxaline derivatives, see: Abasolo *et al.* (1987 ▶); Rodrigo *et al.* (2002 ▶). For inter­esting anti­fungal, herbicidal, anti­dyslipidemic and anti­oxidative activities of quinoxaline derivatives, see: Jampilek *et al.* (2005 ▶); Sashidhara *et al.* (2009 ▶); Watkins *et al.* (2009 ▶).
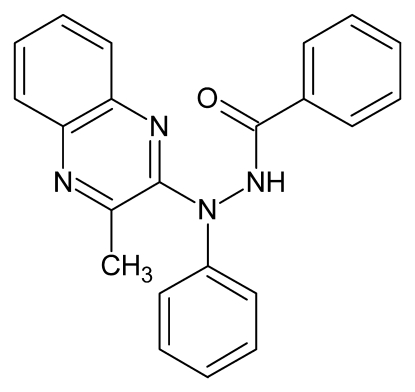


## Experimental

### 

#### Crystal data

C_22_H_18_N_4_O
*M* *_r_* = 354.40
Monoclinic, 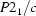

*a* = 18.6809 (12) Å
*b* = 10.5840 (8) Å
*c* = 9.5860 (6) Å
β = 100.108 (3)°
*V* = 1865.9 (2) Å^3^
*Z* = 4
Mo *K*α radiation
μ = 0.08 mm^−1^
*T* = 296 K
0.35 × 0.34 × 0.18 mm

#### Data collection

Bruker APEXII CCD detector diffractometer
19397 measured reflections
4502 independent reflections
2286 reflections with *I* > 2σ(*I*)
*R*_int_ = 0.044

#### Refinement

*R*[*F* ^2^ > 2σ(*F* ^2^)] = 0.048
*wR*(*F* ^2^) = 0.130
*S* = 1.01
4502 reflections
245 parameters
H-atom parameters constrained
Δρ_max_ = 0.13 e Å^−3^
Δρ_min_ = −0.20 e Å^−3^

### 

Data collection: *APEX2* (Bruker, 2005 ▶); cell refinement: *SAINT* (Bruker, 2005 ▶); data reduction: *SAINT*; program(s) used to solve structure: *SHELXS97* (Sheldrick, 2008 ▶); program(s) used to refine structure: *SHELXL97* (Sheldrick, 2008 ▶); molecular graphics: *PLATON* (Spek, 2009 ▶); software used to prepare material for publication: *publCIF* (Westrip, 2010 ▶).

## Supplementary Material

Crystal structure: contains datablocks I, global. DOI: 10.1107/S160053681101703X/bt5532sup1.cif

Structure factors: contains datablocks I. DOI: 10.1107/S160053681101703X/bt5532Isup2.hkl

Supplementary material file. DOI: 10.1107/S160053681101703X/bt5532Isup3.mol

Supplementary material file. DOI: 10.1107/S160053681101703X/bt5532Isup4.cml

Additional supplementary materials:  crystallographic information; 3D view; checkCIF report

## Figures and Tables

**Table 1:** Hydrogen-bond geometry (Å, °) *Cg*1 and *Cg*2 are the centroids of the C1–C6 and C8–C13 rings, respectively.

*D*—H⋯*A*	*D*—H	H⋯*A*	*D*⋯*A*	*D*—H⋯*A*
N4—H6⋯O1^i^	0.86	2.05	2.863 (2)	157
C18—H18⋯O1^ii^	0.93	2.57	3.496 (3)	175
C22—H22*B*⋯*Cg*1^iii^	0.96	2.99	3.696 (2)	131
C20—H20⋯*Cg*2^iv^	0.93	2.94	3.866 (2)	175

Symmetry codes: (i) 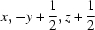; (ii) 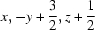; (iii) 
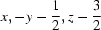
; (iv) 
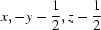
.

**Fig. 1.**
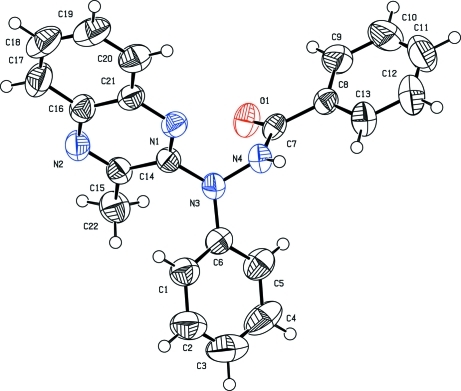
Molecular view of the title compound showing the atom-labeling scheme. Displacement ellipsoids are drawn at the 50% probability level. H atoms are represented as small spheres of arbitrary radii.

**Fig. 2.**
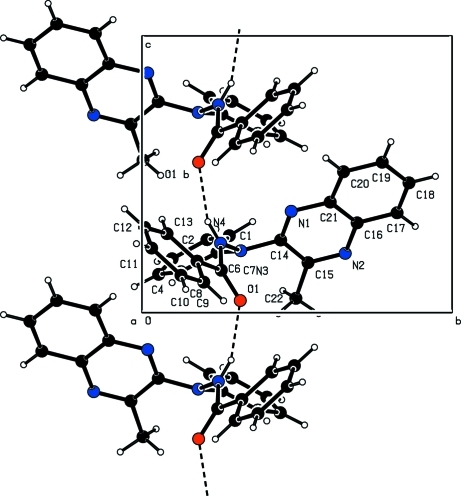
Partial packing view showing the chain formed by N—H···N hydrogen bondings. H atoms not involved in hydrogen bonds have been omitted for clarity

## supplementary crystallographic information

### Comment

Recent advances in targeted therapeutics coupled with new approaches in target identification have accelerated the need to design small compounds with drug like properties. Quinoxaline is well known for its broad coverage in the field of medicine as well as for its application in the pharmaceuticals.

Quinoxaline derivatives were found to exhibit antimicrobial [Kleim *et al.* 1995], antitumor [Abasolo *et al.* 1987], and antituberculous activities [Rodrigo *et al.*2002]. They, also, exhibit interesting antifungal, herbicidal, antidyslipidemic and antioxidative properties [Jampilek *et al.* 2005, Sashidhara *et al.* 2009, Watkins *et al.* 2009].

In the crystal structure of the title compound, the quinoxaline system makes dihedral angles of 86.59 (7) and 63.37 (9) with the benzohydrazide and the phenyl rings, respectively. The benzohydrazide ring makes a dihedral angle of 72.46 (10) with the phenyl ring. The crystal packing is stabilized by N—H···O hydrogen bonds and C—H···π interactions [*Cg*1: (C1 — C2 — C3 — C4 — C5 — C6), and *Cg*2: (C8 — C9 — C10 — C11 — C12 — C13)].

### Experimental

6.5 mmole of 3-methylquinoxalin-2-one are dissolved in 40 ml of THF,8.1 mmol of diphenylnitrileimine and 8.1 mmoles of TEA are added. this mixture solution surmounted by a CaCl2, is refluxed for 24–48 h.After cooling, the salts are removed by filtration and the solvent was evaporated under reduced pressure. The single crystals have been obtained by recrystallization in ethanol.

### Refinement

All H atoms attached to C were fixed geometrically and treated as riding with C—H = 0.96Å (methyl) or 0.93Å (aromatic) with *U*_iso_(H) = 1.2*U*_eq_(C) or 1.5*U*_eq_(methyl).

### Crystal data

**Table:**

C_22_H_18_N_4_O	*F*(000) = 744
*M**_r_* = 354.40	*D*_x_ = 1.262 Mg m^−^^3^
Monoclinic, *P*2_1_/*c*	Mo *K*α radiation, λ = 0.71073 Å
Hall symbol: -P 2ybc	Cell parameters from 345 reflections
*a* = 18.6809 (12) Å	θ = 2.7–26.8°
*b* = 10.5840 (8) Å	µ = 0.08 mm^−^^1^
*c* = 9.5860 (6) Å	*T* = 296 K
β = 100.108 (3)°	Prism, colourless
*V* = 1865.9 (2) Å^3^	0.35 × 0.34 × 0.18 mm
*Z* = 4	

### Data collection

**Table:**

Bruker APEXII CCD detector diffractometer	2286 reflections with *I* > 2σ(*I*)
Radiation source: fine-focus sealed tube	*R*_int_ = 0.044
graphite	θ_max_ = 28.0°, θ_min_ = 1.1°
ω and φ scans	*h* = −24→24
19397 measured reflections	*k* = −8→13
4502 independent reflections	*l* = −12→10

### Refinement

**Table:**

Refinement on *F*^2^	Primary atom site location: structure-invariant direct methods
Least-squares matrix: full	Secondary atom site location: difference Fourier map
*R*[*F*^2^ > 2σ(*F*^2^)] = 0.048	Hydrogen site location: inferred from neighbouring sites
*wR*(*F*^2^) = 0.130	H-atom parameters constrained
*S* = 1.01	*w* = 1/[σ^2^(*F*_o_^2^) + (0.0573*P*)^2^] where *P* = (*F*_o_^2^ + 2*F*_c_^2^)/3
4502 reflections	(Δ/σ)_max_ < 0.001
245 parameters	Δρ_max_ = 0.13 e Å^−^^3^
0 restraints	Δρ_min_ = −0.20 e Å^−^^3^

### Special details

**Table:**

Experimental. The data collection nominally covered a sphere of reciprocal space, by a combination of two sets of exposures; each set had a different φ angle for the crystal and each exposure covered 0.5° in ω and 30 s in time. The crystal-to-detector distance was 37.5 mm.
Geometry. All e.s.d.'s (except the e.s.d. in the dihedral angle between two l.s. planes) are estimated using the full covariance matrix. The cell e.s.d.'s are taken into account individually in the estimation of e.s.d.'s in distances, angles and torsion angles; correlations between e.s.d.'s in cell parameters are only used when they are defined by crystal symmetry. An approximate (isotropic) treatment of cell e.s.d.'s is used for estimating e.s.d.'s involving l.s. planes.
Refinement. Refinement of *F*^2^ against ALL reflections. The weighted *R*-factor *wR* and goodness of fit *S* are based on *F*^2^, conventional *R*-factors *R* are based on *F*, with *F* set to zero for negative *F*^2^. The threshold expression of *F*^2^ > σ(*F*^2^) is used only for calculating *R*-factors(gt) *etc*. and is not relevant to the choice of reflections for refinement. *R*-factors based on *F*^2^ are statistically about twice as large as those based on *F*, and *R*- factors based on ALL data will be even larger.

### Fractional atomic coordinates and isotropic or equivalent isotropic displacement parameters (Å^2^)

**Table:**

	*x*	*y*	*z*	*U*_iso_*/*U*_eq_	
N1	0.26330 (7)	0.48128 (13)	0.36620 (14)	0.0485 (4)	
N2	0.16619 (8)	0.65538 (14)	0.21427 (16)	0.0568 (4)	
N3	0.20960 (7)	0.31873 (13)	0.22361 (14)	0.0480 (4)	
O1	0.30401 (6)	0.31707 (11)	0.04356 (12)	0.0578 (3)	
C8	0.38609 (8)	0.17788 (14)	0.18621 (16)	0.0439 (4)	
C7	0.31928 (8)	0.25585 (14)	0.15321 (16)	0.0415 (4)	
C21	0.26800 (9)	0.60703 (16)	0.39925 (18)	0.0494 (4)	
N4	0.27553 (7)	0.25367 (12)	0.25080 (13)	0.0465 (3)	
H6	0.2880	0.2128	0.3288	0.056*	
C14	0.21230 (8)	0.44722 (15)	0.26227 (17)	0.0437 (4)	
C15	0.16273 (8)	0.53545 (17)	0.18063 (17)	0.0488 (4)	
C16	0.21862 (9)	0.69384 (16)	0.32436 (19)	0.0524 (4)	
C6	0.14703 (9)	0.24218 (16)	0.21688 (17)	0.0483 (4)	
C20	0.32204 (10)	0.65006 (19)	0.5091 (2)	0.0669 (5)	
H20	0.3549	0.5931	0.5588	0.080*	
C13	0.39464 (10)	0.08112 (17)	0.28516 (18)	0.0595 (5)	
H13	0.3579	0.0650	0.3368	0.071*	
C22	0.11033 (9)	0.49634 (18)	0.05163 (19)	0.0671 (5)	
H22A	0.0695	0.4544	0.0797	0.101*	
H22B	0.1340	0.4398	−0.0040	0.101*	
H22C	0.0937	0.5697	−0.0036	0.101*	
C17	0.22522 (11)	0.82237 (18)	0.3608 (2)	0.0722 (6)	
H17	0.1934	0.8811	0.3116	0.087*	
C1	0.08899 (9)	0.28171 (18)	0.27791 (19)	0.0597 (5)	
H1	0.0921	0.3569	0.3288	0.072*	
C19	0.32655 (12)	0.7747 (2)	0.5432 (2)	0.0783 (6)	
H19	0.3621	0.8024	0.6172	0.094*	
C9	0.44130 (10)	0.19941 (18)	0.1104 (2)	0.0680 (5)	
H9	0.4366	0.2633	0.0428	0.082*	
C5	0.14310 (10)	0.12638 (18)	0.14787 (18)	0.0647 (5)	
H5	0.1821	0.0976	0.1083	0.078*	
C12	0.45673 (13)	0.0085 (2)	0.3081 (2)	0.0784 (6)	
H12	0.4617	−0.0562	0.3747	0.094*	
C18	0.27851 (12)	0.8610 (2)	0.4687 (2)	0.0800 (7)	
H18	0.2826	0.9462	0.4925	0.096*	
C3	0.02217 (13)	0.0973 (3)	0.1942 (3)	0.0912 (7)	
H3	−0.0202	0.0496	0.1845	0.109*	
C11	0.51074 (12)	0.0315 (2)	0.2334 (3)	0.0920 (8)	
H11	0.5527	−0.0174	0.2492	0.110*	
C2	0.02629 (11)	0.2097 (2)	0.2635 (2)	0.0786 (6)	
H2	−0.0132	0.2385	0.3015	0.094*	
C4	0.08056 (14)	0.0540 (2)	0.1384 (2)	0.0846 (7)	
H4	0.0781	−0.0244	0.0940	0.102*	
C10	0.50361 (11)	0.1261 (2)	0.1350 (3)	0.0936 (7)	
H10	0.5408	0.1413	0.0842	0.112*	

### Atomic displacement parameters (Å^2^)

**Table:**

	*U*^11^	*U*^22^	*U*^33^	*U*^12^	*U*^13^	*U*^23^
N1	0.0488 (8)	0.0440 (9)	0.0508 (9)	−0.0024 (6)	0.0038 (7)	−0.0003 (7)
N2	0.0587 (10)	0.0500 (9)	0.0627 (10)	0.0109 (7)	0.0132 (8)	0.0060 (8)
N3	0.0438 (8)	0.0416 (8)	0.0578 (9)	0.0045 (6)	0.0069 (6)	−0.0038 (7)
O1	0.0811 (9)	0.0501 (7)	0.0434 (7)	0.0102 (6)	0.0140 (6)	0.0074 (6)
C8	0.0494 (10)	0.0392 (9)	0.0430 (9)	0.0002 (7)	0.0078 (8)	−0.0100 (8)
C7	0.0550 (10)	0.0329 (8)	0.0367 (9)	−0.0027 (7)	0.0085 (8)	−0.0046 (7)
C21	0.0529 (11)	0.0451 (10)	0.0525 (10)	−0.0055 (8)	0.0155 (9)	−0.0019 (9)
N4	0.0519 (8)	0.0461 (8)	0.0425 (8)	0.0109 (7)	0.0108 (6)	0.0039 (6)
C14	0.0434 (9)	0.0433 (10)	0.0463 (10)	0.0012 (7)	0.0130 (8)	−0.0007 (8)
C15	0.0464 (10)	0.0515 (11)	0.0491 (10)	0.0040 (8)	0.0102 (8)	0.0025 (8)
C16	0.0593 (11)	0.0452 (11)	0.0568 (11)	0.0018 (8)	0.0211 (9)	−0.0004 (9)
C6	0.0535 (11)	0.0469 (10)	0.0420 (9)	−0.0038 (8)	0.0014 (8)	0.0021 (8)
C20	0.0673 (13)	0.0631 (13)	0.0680 (13)	−0.0137 (10)	0.0057 (10)	−0.0070 (10)
C13	0.0703 (12)	0.0569 (12)	0.0530 (11)	0.0181 (9)	0.0151 (9)	0.0026 (9)
C22	0.0625 (12)	0.0742 (14)	0.0594 (12)	0.0054 (10)	−0.0036 (9)	0.0084 (10)
C17	0.0926 (16)	0.0447 (12)	0.0864 (15)	0.0049 (10)	0.0350 (13)	−0.0032 (11)
C1	0.0585 (12)	0.0592 (12)	0.0619 (12)	−0.0044 (9)	0.0119 (9)	0.0024 (9)
C19	0.0889 (16)	0.0694 (16)	0.0789 (15)	−0.0287 (12)	0.0211 (12)	−0.0173 (12)
C9	0.0633 (13)	0.0635 (13)	0.0811 (14)	−0.0031 (10)	0.0237 (11)	−0.0011 (11)
C5	0.0803 (14)	0.0571 (12)	0.0537 (11)	−0.0069 (10)	0.0030 (10)	−0.0043 (10)
C12	0.0894 (16)	0.0696 (15)	0.0717 (14)	0.0300 (12)	0.0017 (13)	−0.0025 (11)
C18	0.1086 (18)	0.0520 (13)	0.0909 (17)	−0.0234 (13)	0.0488 (15)	−0.0208 (13)
C3	0.0790 (17)	0.101 (2)	0.0875 (17)	−0.0368 (14)	−0.0014 (13)	0.0133 (15)
C11	0.0630 (15)	0.0860 (18)	0.119 (2)	0.0220 (13)	−0.0057 (14)	−0.0242 (16)
C2	0.0630 (14)	0.0899 (17)	0.0824 (15)	−0.0134 (12)	0.0112 (11)	0.0153 (13)
C4	0.1145 (19)	0.0618 (14)	0.0688 (14)	−0.0309 (14)	−0.0081 (13)	−0.0045 (11)
C10	0.0595 (14)	0.0962 (19)	0.133 (2)	−0.0001 (13)	0.0396 (14)	−0.0130 (17)

### Geometric parameters (Å, °)

**Table:**

N1—C14	1.3028 (19)	C22—H22A	0.9600
N1—C21	1.367 (2)	C22—H22B	0.9600
N2—C15	1.308 (2)	C22—H22C	0.9600
N2—C16	1.370 (2)	C17—C18	1.366 (3)
N3—N4	1.3951 (16)	C17—H17	0.9300
N3—C14	1.408 (2)	C1—C2	1.384 (2)
N3—C6	1.414 (2)	C1—H1	0.9300
O1—C7	1.2251 (17)	C19—C18	1.388 (3)
C8—C9	1.381 (2)	C19—H19	0.9300
C8—C13	1.386 (2)	C9—C10	1.385 (3)
C8—C7	1.483 (2)	C9—H9	0.9300
C7—N4	1.3461 (18)	C5—C4	1.387 (3)
C21—C20	1.401 (2)	C5—H5	0.9300
C21—C16	1.406 (2)	C12—C11	1.358 (3)
N4—H6	0.8600	C12—H12	0.9300
C14—C15	1.445 (2)	C18—H18	0.9300
C15—C22	1.495 (2)	C3—C2	1.358 (3)
C16—C17	1.405 (2)	C3—C4	1.375 (3)
C6—C1	1.384 (2)	C3—H3	0.9300
C6—C5	1.389 (2)	C11—C10	1.366 (3)
C20—C19	1.358 (3)	C11—H11	0.9300
C20—H20	0.9300	C2—H2	0.9300
C13—C12	1.376 (2)	C4—H4	0.9300
C13—H13	0.9300	C10—H10	0.9300
			
C14—N1—C21	117.07 (14)	H22A—C22—H22C	109.5
C15—N2—C16	118.43 (14)	H22B—C22—H22C	109.5
N4—N3—C14	115.97 (12)	C18—C17—C16	119.9 (2)
N4—N3—C6	114.91 (13)	C18—C17—H17	120.0
C14—N3—C6	123.80 (13)	C16—C17—H17	120.0
C9—C8—C13	118.36 (16)	C2—C1—C6	120.24 (19)
C9—C8—C7	118.30 (16)	C2—C1—H1	119.9
C13—C8—C7	123.28 (15)	C6—C1—H1	119.9
O1—C7—N4	121.80 (14)	C20—C19—C18	120.6 (2)
O1—C7—C8	122.53 (14)	C20—C19—H19	119.7
N4—C7—C8	115.66 (14)	C18—C19—H19	119.7
N1—C21—C20	119.94 (17)	C8—C9—C10	120.1 (2)
N1—C21—C16	120.52 (16)	C8—C9—H9	120.0
C20—C21—C16	119.53 (17)	C10—C9—H9	120.0
C7—N4—N3	119.04 (13)	C4—C5—C6	119.50 (19)
C7—N4—H6	120.5	C4—C5—H5	120.3
N3—N4—H6	120.5	C6—C5—H5	120.3
N1—C14—N3	117.14 (14)	C11—C12—C13	120.0 (2)
N1—C14—C15	123.27 (15)	C11—C12—H12	120.0
N3—C14—C15	119.46 (14)	C13—C12—H12	120.0
N2—C15—C14	119.62 (15)	C17—C18—C19	120.8 (2)
N2—C15—C22	118.00 (15)	C17—C18—H18	119.6
C14—C15—C22	122.24 (16)	C19—C18—H18	119.6
N2—C16—C17	119.95 (17)	C2—C3—C4	120.1 (2)
N2—C16—C21	121.02 (16)	C2—C3—H3	119.9
C17—C16—C21	118.99 (18)	C4—C3—H3	119.9
C1—C6—C5	119.22 (17)	C12—C11—C10	120.3 (2)
C1—C6—N3	120.67 (15)	C12—C11—H11	119.9
C5—C6—N3	120.11 (16)	C10—C11—H11	119.9
C19—C20—C21	120.2 (2)	C3—C2—C1	120.4 (2)
C19—C20—H20	119.9	C3—C2—H2	119.8
C21—C20—H20	119.9	C1—C2—H2	119.8
C12—C13—C8	120.95 (19)	C3—C4—C5	120.5 (2)
C12—C13—H13	119.5	C3—C4—H4	119.8
C8—C13—H13	119.5	C5—C4—H4	119.8
C15—C22—H22A	109.5	C11—C10—C9	120.4 (2)
C15—C22—H22B	109.5	C11—C10—H10	119.8
H22A—C22—H22B	109.5	C9—C10—H10	119.8
C15—C22—H22C	109.5		
			
C21—N1—C14—N3	177.02 (16)	O1—C7—C8—C9	16.7 (3)
C21—N1—C14—C15	1.5 (3)	O1—C7—C8—C13	−160.64 (18)
C14—N1—C21—C16	0.9 (3)	N4—C7—C8—C9	−164.24 (18)
C14—N1—C21—C20	−179.40 (18)	N4—C7—C8—C13	18.4 (3)
C16—N2—C15—C14	1.8 (3)	C7—C8—C9—C10	−178.0 (2)
C16—N2—C15—C22	−174.01 (17)	C13—C8—C9—C10	−0.5 (3)
C15—N2—C16—C17	178.2 (2)	C7—C8—C13—C12	177.6 (2)
C15—N2—C16—C21	0.4 (3)	C9—C8—C13—C12	0.3 (3)
C6—N3—N4—C7	117.98 (18)	C8—C9—C10—C11	0.4 (4)
C14—N3—N4—C7	−86.8 (2)	C9—C10—C11—C12	−0.1 (5)
N4—N3—C6—C1	138.71 (18)	C10—C11—C12—C13	−0.2 (5)
N4—N3—C6—C5	−41.6 (2)	C11—C12—C13—C8	0.1 (4)
C14—N3—C6—C1	−14.3 (3)	N1—C14—C15—N2	−2.9 (3)
C14—N3—C6—C5	165.36 (18)	N1—C14—C15—C22	172.71 (17)
N4—N3—C14—N1	−27.4 (2)	N3—C14—C15—N2	−178.39 (17)
N4—N3—C14—C15	148.32 (16)	N3—C14—C15—C22	−2.8 (3)
C6—N3—C14—N1	125.33 (19)	N2—C16—C17—C18	−178.6 (2)
C6—N3—C14—C15	−58.9 (2)	C21—C16—C17—C18	−0.8 (3)
N3—N4—C7—O1	2.7 (3)	N2—C16—C21—N1	−1.9 (3)
N3—N4—C7—C8	−176.34 (15)	N2—C16—C21—C20	178.37 (19)
C6—C1—C2—C3	2.7 (4)	C17—C16—C21—N1	−179.65 (19)
C2—C1—C6—N3	176.1 (2)	C17—C16—C21—C20	0.6 (3)
C2—C1—C6—C5	−3.6 (3)	C16—C17—C18—C19	0.0 (4)
C1—C2—C3—C4	0.1 (4)	C17—C18—C19—C20	1.0 (4)
C2—C3—C4—C5	−2.1 (4)	C18—C19—C20—C21	−1.2 (4)
C3—C4—C5—C6	1.2 (4)	C19—C20—C21—N1	−179.4 (2)
C4—C5—C6—N3	−178.0 (2)	C19—C20—C21—C16	0.4 (3)
C4—C5—C6—C1	1.6 (3)		

### Hydrogen-bond geometry (Å, °)

**Table:**

Cg1 and Cg2 are the centroids of the C1–C6 and C8–C13 rings, respectively.

**Table:**

*D*—H···*A*	*D*—H	H···*A*	*D*···*A*	*D*—H···*A*
N4—H6···O1^i^	0.86	2.05	2.863 (2)	157.
C18—H18···O1^ii^	0.93	2.57	3.496 (3)	175.
C22—H22B···Cg1^iii^	0.96	2.99	3.696 (2)	131
C20—H20···Cg2^iv^	0.93	2.94	3.866 (2)	175

Symmetry codes: (i) *x*, −*y*+1/2, *z*+1/2; (ii) *x*, −*y*+3/2, *z*+1/2; (iii) *x*, −*y*−1/2, *z*−3/2; (iv) *x*, −*y*−1/2, *z*−1/2.
